# Big bottlenecks in cardiovascular tissue engineering

**DOI:** 10.1038/s42003-018-0202-8

**Published:** 2018-11-21

**Authors:** Ngan F. Huang, Vahid Serpooshan, Viola B. Morris, Nazish Sayed, Gaspard Pardon, Oscar J. Abilez, Karina H. Nakayama, Beth L. Pruitt, Sean M. Wu, Young-sup Yoon, Jianyi Zhang, Joseph C. Wu

**Affiliations:** 1 Stanford Cardiovascular Institute, Stanford University School of Medicine, Stanford, 94305 CA USA; 2 Department of Cardiothoracic Surgery, Stanford University School of Medicine, Stanford, 94305 CA USA; 3 Veteran Affairs Palo Alto Health Care System, Palo Alto, 94304 CA USA; 4 Wallace H. Coulter Department of Biomedical Engineering, Emory University and Georgia Institute of Technology, Atlanta, 30332 GA USA; 5 Department of Pediatrics, Emory University School of Medicine, Atlanta, 30307 GA USA; 6 Department of Medicine, Division of Cardiology, Emory University, Atlanta, 30307 GA USA; 7 Department of Bioengineering, Stanford University, Stanford, 94305 CA USA; 8 Department of Mechanical Engineering, Stanford University, Stanford, 94305 CA USA; 9 Departments of Mechanical Engineering; BioMolecular Science and Engineering; and Molecular, Cellular and Developmental Biology, University of California at Santa Barbara, Santa Barbara, 93106 CA USA; 10 Division of Cardiovascular Medicine, Department of Medicine, Stanford University School of Medicine, Stanford, 94305 CA USA; 11 Institute for Stem Cell Biology and Regenerative Medicine, Stanford University, Stanford, 94305 CA USA; 12 Department of Bioengineering, School of Medicine, University of Alabama at Birmingham, Birmingham, 35294 AL USA

## Abstract

Although tissue engineering using human-induced pluripotent stem cells is a promising approach for treatment of cardiovascular diseases, some limiting factors include the survival, electrical integration, maturity, scalability, and immune response of three-dimensional (3D) engineered tissues. Here we discuss these important roadblocks facing the tissue engineering field and suggest potential approaches to overcome these challenges.

## Abstract (web summary)

In this Comment, Ngan Huang et al. discuss recent advances in cardiovascular tissue engineering and some of the main challenges that remain in translating these advances to the clinic. The authors propose future direction for the field to focus research efforts.

## Introduction

Cardiovascular diseases are the leading cause of heart failure and mortality in the United States, and heart transplant remains the most viable and effective option for treatment^1^. However, a major drawback for heart transplantation is the chronic shortage of donor organs and tissues. Furthermore, heart transplant recipients face serious challenges in long-term survival in the form of adverse effects of immunosuppression and chronic immune rejection^2^. Accordingly, there is a compelling need for alternative strategies to improve the management of heart failure. Tissue engineering—a multi-disciplinary approach that combines life sciences and engineering to manufacture functional tissue equivalents, such as engineered myocardial tissue—is emerging as a promising alternative to organ replacement or mechanical support^3^.

**Fig. 1:**
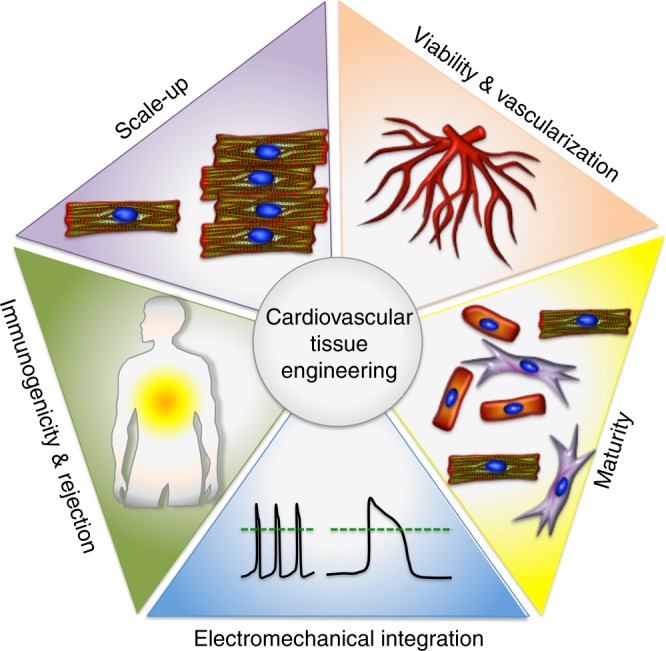
Bottlenecks in cardiovascular tissue engineering. These challenges include the survival, electrical integration, maturity, scalability, and immune response of three-dimensional engineered tissues

Owing to the generally non-proliferative nature of contractile cardiomyocytes (CM) in the myocardium, the efficient generation of CMs from human-induced pluripotent stem cells (hiPSCs)^4^ has been a major advancement in cardiovascular tissue engineering^5^. Although some reports have demonstrated the efficacy of hiPSC-CM-derived engineered myocardial tissue in small and large preclinical animal models of heart failure^6–10^, challenges exist that hinder the successful clinical application of hiPSC-CM-derived engineered myocardial tissue. These include the survival, electrical integration, and immune response of scalable three-dimensional (3D) engineered tissues, as well as issues concerning the maturity and function of hiPSC-CMs (Fig. 1). Below we discuss some of the important roadblocks facing this field, and the potential approaches to overcome these challenges.

## How can we generate clinically relevant numbers of hiPSC-CMs for engineering myocardial tissue?

The human myocardium consists of ~10^9^ cells, among which CMs comprise about one-third of the total cells. The ability to generate such a large number of hiPSC-CMs for tissue engineering remains a challenge. Although highly efficient differentiation protocols can now produce on the order of ~10^7^ cells in a single dish, scaling up to 10^9^ cells would require nearly 100 dishes. Nevertheless, recent reports using 10-layer 1.2 L culture flasks demonstrate the feasibility of generating a clinically relevant number of 10^9^ cells with >60% purity of hiPSC-CMs^11^. An alternative approach for scaling up the number of hiPSC-CMs in a more space-efficient and cost-effective manner is 3D suspension differentiation platforms. One example is microcarriers, which are materials that remain suspended in cell culture medium in a culture vessel and support cellular attachment. Owing to their large surface area per volume, microcarriers can facilitate the attachment and differentiation of hiPSCs^12^. Another example is the use of 3D aggregates of hiPSCs, which can be differentiated in suspension culture, achieving ~10^9^ hiPSC-CMs in a 1 L spinner flask^13^. The next step toward generating clinically relevant numbers of CMs would be to engineer myocardial tissues (>10 cm × 10 cm) with a physiologically relevant cell density (~10^8^/cm^3^)^14^ that consist of hiPSC-CMs in co-culture with support cells that comprise the remaining two-thirds of the myocardium (i.e., endothelial cells, pericytes, or fibroblasts) to promote intercellular interactions capable of sustaining the function and phenotype of hiPSC-CMs^15^. Future steps will also include the development of efficient suspension differentiation protocols for specific subtypes of hiPSC-CMs (i.e., atrial, ventricular, nodal, and Purkinje), because most differentiation protocols have been optimized to predominantly produce ventricular hiPSC-CMs^16–18^. Further development may make microcarriers more amenable to generating clinically relevant numbers of hiPSC-CMs in co-culture with vascular support cells.

## How can we maintain the viability of 3D engineered myocardial tissues?

A major hurdle for the survival of 3D engineered tissues is poor perfusion of nutrients^19^. Whereas the typical inter-capillary distance in the myocardium is ~20 μm^20^, the thickness of 3D engineered myocardial tissue spans mm-to-cm thicknesses. Without a reliable method to transport nutrients and oxygen throughout the engineered tissue, the cells embedded in the tissue construct do not remain viable over time. Consequently, perfusion of the engineered myocardial tissue is critical for long-term tissue survival^21^. Although bioreactors can maintain the viability of engineered myocardial tissues in vitro by active perfusion^22^, in the absence of a pre-existing in vitro vascular network to integrate the engineered tissue with the host vasculature upon transplantation, cell viability is not sustainable in vivo. Vascularization of engineered myocardial tissue can be achieved by the induction of angiogenic molecules, cell–cell interactions, or mechanical factors^23^. Co-culture of hiPSC-CMs with endothelial cells or endothelial progenitor cells can form primitive vessel-like structures with the potential for in vivo anastomosis^10^. However, for greater control of the vessel architecture, techniques such as 3D bioprinting^24^, micropatterning^25^, and microfluidic systems^26^ have been shown to be beneficial for anastomosis and tissue integration. Among these approaches, 3D bioprinting has been particularly promising, but it is currently limited by inadequate bioinks and multi-material bioprinting modalities needed for creation of cell-laden, 3D vascular constructs that maintain tissue-mimetic stiffness, cell density, and function^24,27^. The next important step will be the creation of vascularized 3D engineered myocardial constructs that are perfusable both in vitro and in vivo, and supportive of cardiac muscle maturity and global contractile function^28^. An ongoing competition from the National Aeronautics and Space Administration (NASA) and the New Organ Alliance seeks to overcome the vascularization challenge by awarding a $500K prize to teams that successfully engineer functionally vascularized tissues^29^.

## How can we achieve functional integration between engineered cardiovascular tissue and host myocardium?

In addition to the low engraftment rate being one of the first major roadblocks, another important hurdle with engineered myocardial tissue therapy is the electromechanical integration between the transplanted engineered myocardial tissue and the host myocardium. Because engineered myocardial tissue may possess greater heterogeneity in cellular organization than native tissues, reentry arrhythmia/block is a significant concern^30^. When the electrical wave fronts transit from the native myocardium to the 3D engineered myocardial tissue, passing through a fibrotic interface or vice-versa, a block of the wave front can be potentially life-threatening. Such a block may result from the heterogeneity of electrophysiological parameters, such as action potential duration or excitability. To minimize the risk of arrhythmia, recent advances in conductive scaffolds could help improve electrical communication between the engineered myocardial tissue implants and the host myocardium^31–34^. Furthermore, as epicardial patches are physically separated from the host myocardium, which hinders electrical coupling, approaches to recruit epicardial cells to the engineered myocardial tissue using bioactive peptides^35^ is a promising approach. These strategies will help provide engineered myocardial tissue with electromechanical characteristics equivalent to that of the naive cardiac myocardium.

## How can we improve the maturity and function of engineered myocardial tissue composed of hiPSC-CMs?

Although highly efficient protocols for hiPSC-CM generation have greatly accelerated the pace of cardiovascular tissue engineering discoveries^36–38^, these protocols yield largely an immature cell population with variability in functions and structures. For instance, whereas primary adult CMs are morphologically rectangular in shape with distinctive electrical and mechanical properties, hiPSC-CMs generally are more amorphous in shape, with electrical and mechanical properties more resembling those of embryonic CMs. To engineer adult-like mature and functional engineered myocardial tissue, the heterogeneity and immaturity of hiPSC-CMs must be addressed. Mechanical factors have been shown to improve the maturity and function of hiPSC-CMs. For example, spatially patterned substrates with physiological stiffness (6–10 kPa) that impart a 7:1 aspect ratio in cell shape have been shown to increase hiPSC-CM contractility and enhance calcium handling and electrophysiology, thereby producing more mature and aligned sarcomere organization^39,40^. Mechanical strain stimulation of early-stage hiPSC-CMs with increasing intensity over time can also impact adult-like gene expression, sarcomeric length, and ultrastructure^41^. These studies underscore the importance of mechanical factors in enhancing hiPSC-CM maturity and function. Because these findings have been reported only in relative small engineered myocardial tissues, the next step will be to translate these approaches using larger 3D engineered myocardial tissues in large animal disease models.

## How can we overcome rejection of engineered myocardial tissue after transplantation in vivo?

A major roadblock to the application of hiPSC-based therapies is immune rejection by the host^42^. Despite controversies surrounding the immunogenicity of hiPSC derivatives, almost all studies that involve transplantation of hiPSC-CMs induce immunosuppression in their animal models^9,43^. As a solution, there is a compelling need to advance the hiPSC technology using off-the-shelf sources of cardiovascular cells and development of tissue sources. Although human leukocyte antigen (HLA)-matched hiPSC tissue banks could be a valuable source of tissues for personalized therapeutics and an effective way to deliver cell therapy to a large number of patients^44^, a lack of basic understanding in the complexities of ethnic diversity is a challenge. Probabilistic models show that a bank of hiPSCs generated from 100 of the most prevalent HLA types would be a haplotype match for 78% of Europeans, 63% of Asians, and 45% of African Americans^45^, suggesting that the development of an allogeneic cell bank may be potentially feasible for relatively ethnically homogenous countries, but challenging for diverse ones. Moreover, such an endeavor would require a concerted effort by international groups to create a sufficient tissue repository^45^. Finally, even HLA-matched tissues are theoretically capable of triggering an immune rejection that would still require immunosuppression. Consequently, recent efforts aim to genetically engineer so-called master hiPSC lines that give rise to immune-tolerant hiPSC derivatives^46^. These universal off-the-shelf hiPSC derivatives can be generated by introducing multiple modalities that include immune evasion (by deleting HLA) and immune suppression (by overexpressing immunosuppressive proteins). Importantly, these HLA-null master hiPSCs could eventually be used to engineer a hypo-immunogenic cardiac patch as an off-the-shelf product that can be used universally for cardiac repair. Alternatively, engineering approaches may one day create allogeneic hiPSC derivatives that escape immune rejection. Allogenic hiPSCs could have far-reaching applications such as generating ready-to-use engineered myocardial tissue for therapeutic transplantation.

## Future outlook

To date, the engineering of myocardial tissue for regenerative medicine has been greatly advanced by the use of hiPSCs, biocompatible materials, and the control of mechanical properties. However, besides these five bottlenecks, other important challenges that need to be addressed include cryopreservation of 3D engineered myocardium, attainment of functional cardiovascular tissue in vitro, the recapitulation of native cell–cell interactions between hiPSC-CMs and support cells within the engineered tissues, and development of cost-effective manufacturing processes for scaling up.

In the future, we anticipate increased use of microphysiological systems for high-throughput optimization of cellular composition, geometry, and paracrine factors to maximize the survival and function of engineered myocardial tissue. The aim of this microscale approach is to minimize the number of cells and reagents needed to determine optimal properties in engineered myocardial tissues. To accelerate clinical translation, engineered myocardial tissues derived from HLA-null lines will be further developed to be amenable to cryopreservation, enabling a true off-the-shelf product. With the goal of reducing the costs and time associated with regulatory approval, countries like Japan have recently adopted policies that conditionally approve hiPSC-based experimental therapies in humans based on limited clinical safety data, and allowing to up to 7 years for researchers to provide further evidence of safety and efficacy. Such policies enable clinical testing to be performed more expeditiously without the need for comprehensive data analysis before clinical testing^47^. As the generation of hiPSCs becomes routine using safe reprogramming approaches that prevent unintended genomic integration, hiPSC derivatives will gain further traction for clinical translation.

We envision a future in which patients who are diagnosed with heart failure will simply be prescribed a cryopreserved, immune-tolerant engineered myocardium composed of hiPSC-CMs and other support cells that comprise the myocardium. With the rapid progress in new technologies and continuing refinement of protocols being worked on by a large international community of active researchers in this field, this future is well within our reach and will benefit millions of heart disease patients.
